# The 6th Qatar International Internal Medicine (6-QIIM) Conference 2025: Enhancing patient care through regional and international collaboration and innovation

**DOI:** 10.5339/qmj.2025.1

**Published:** 2025-03-18

**Authors:** Abdel-Naser Elzouki, Mohamad M. Alkadi, Raza Akbar, Joe Mathew, Muhammad Zahid, Abdullatif Alkhal, Ahmed Al-Mohammed

**Affiliations:** ^1^Department of Medicine, Hamad Medical Corporation, Doha, Qatar; ^2^College of Medicine, Qatar University, Doha, Qatar; ^3^Weill Cornell Medicine-Qatar, Doha, Qatar *Correspondence: Abdel-Naser Elzouki. Email: aelzouki@hamad.qa

Internal Medicine has witnessed remarkable advancements in recent years, encompassing telemedicine, personalized medicine, and artificial intelligence. However, significant challenges persist. Non-communicable diseases such as diabetes mellitus, hypertension, dyslipidemia, and cancer place immense strain on healthcare systems. Furthermore, antimicrobial resistance poses a silent yet growing threat. Additionally, the rising global aging population necessitates the development of innovative approaches to effectively manage the growing prevalence of patients with complex, multi-morbidity conditions. Addressing these challenges requires a coordinated, evidence-based approach, reinforced by international collaboration and interdisciplinary research.

Hamad Medical Corporation (HMC) stands as a leading center of excellence in clinical care, education, and research in Qatar and the surrounding region, regularly organizing accredited continuous professional development events of varying scales and focuses. The Internal Medicine Department at HMC hosted the 6th Qatar International Internal Medicine (6-QIIM) Conference in Doha, Qatar, from January 30 to February 1, 2025. This event attracted over 1,400 participants from Qatar and 15 other countries. The conference reflected HMC's shared mission, values, and interests with local, regional, and global partners, including the American College of Physicians (ACP) and the Royal College of Physicians (RCP) of the United Kingdom, aimed at promoting excellence in clinical practice, medical education, and research.

In line with HMC's commitment to excellence in patient care, the 6-QIIM Conference 2025 served as a premier scientific platform to explore current key issues in internal medicine and its related subspecialties. The conference's educational objectives included delivering insights into “state-of-the-art advancements in internal medicine practice”. These objectives were achieved through a series of interactive sessions and workshops. The conference featured distinguished plenary and keynote presentations from renowned international and regional speakers who addressed critical themes. In-depth symposia delved into specialized topics pertinent to both inpatient and outpatient internal medicine practices. Participants also had access to a range of workshops that included hands-on training in medical procedures and POCUS (Point-of-Care Ultrasound), as well as discussions on challenging ECG cases, comprehensive diabetes care, obesity management in clinical practice, and practical guidance on antibiotic prescribing. Other sessions focused on strategies for succeeding in the MRCP-PACES and internal medicine board examinations, along with insights into clinical research trial design and analysis, as well as medical writing. Additionally, the “Meet the Expert” sessions provided learners with the opportunity to interact directly with leading local, regional, and international experts to discuss common clinical scenarios in internal medicine. On the second day, the ACP “Doctor's Dilemma” competition fostered a lively and competitive environment, featuring teams from four internal medicine residency programs across the Gulf region – Qatar, the Kingdom of Saudi Arabia, Oman, and the United Arab Emirates – who competed against one another.

Over the course of the three-day event, the 6-QIIM Conference 2025 highlighted the contributions of esteemed faculty and speakers from national institutions such as HMC, Qatar University, and Weill Cornell Medicine-Qatar, as well as representatives from international organizations, including the ACP and the RCP of the United Kingdom. These academic collaborations reinforced a commitment to scientific excellence and continuous professional development. The conference witnessed an exceptional turnout and active engagement, reflecting the dedication of healthcare professionals to advancing internal medicine and improving healthcare outcomes. Participants engaged in meaningful discussions, exchanged valuable insights, and fostered professional relationships, laying the foundation for future collaborations and innovations in patient care.

Over 300 abstracts on current research and clinical practices in Qatar and worldwide were submitted to the conference. Of these, 250 were accepted for presentation as free communications and posters. A dedicated judging committee selected the top six submissions, which were awarded during the conference and designated as “Award Recipient”. The accepted abstracts have been published in the current issue of *Qatar Medical Journal* as a supplementary file.

Physicians are dedicated lifelong learners and conferences such as the 6-QIIM Conference 2025 offer essential opportunities for sharing insights, debating emerging evidence, and refining clinical skills. Yet, beyond clinical expertise, physicians must embrace leadership roles. Clinicians are uniquely positioned to advocate systemic change and champion policies that enhance healthcare services and improve patient outcomes.

